# Medication Adherence in Patients With Uncontrolled Hypertension & Hypertensive Crisis Presenting to a Hospital Setting in Karachi, Pakistan

**DOI:** 10.7759/cureus.33995

**Published:** 2023-01-20

**Authors:** Fatimah S Yousuf, Muhammad Arbaz A Khan, Raheela Bibi, Aiman Arif, Ainan Arshad, Aysha Almas

**Affiliations:** 1 Medicine, Aga Khan University Hospital, Karachi, PAK; 2 Medicine, Aga Khan University Medical College, Karachi, PAK

**Keywords:** hypertension, anxiety, hypertensive crisis, medication adherence, frequency

## Abstract

Background: Hypertension is an established risk factor for cardiovascular disease. Non-adherence to antihypertensive medications contributes to poorly controlled hypertension while also increasing the risk of hypertensive crisis. The aim of our study was thus to estimate the frequency of adherence to antihypertensive medications in our population and also see if an association exists between adherence and the development of a hypertensive crisis.

Methods: This cross-sectional study was undertaken on patients admitted to Aga Khan University Hospital, Karachi, Pakistan, between July 2020 and March 2022. All patients with uncontrolled hypertension with systolic blood pressure >140 and diastolic blood pressure >90 who were admitted through the emergency department were included. A systolic blood pressure over 180mmHg or diastolic blood pressure over 120mmHg, with or without end-organ damage was used to define a hypertensive crisis. Adherence to medications was assessed using the 4-item Morisky Green Levine (MGL) scale. Each item was scored as 1 and then added together to get a final score out of 4 with a score of ≥3 signifying adherence while a cumulative score of 0, 1, or 2 was classified as non-adherence.

Results: We found that 64% of the cohort were adherent to their medications while 93 (36%) were non-adherent. The most common comorbid condition was found to be diabetes mellitus (54.8%). Around 146 (64.6%) patients were on a single anti-hypertensive agent. Depression as calculated according to the hospital anxiety and depression scale (HADS) was seen in 133 (51.2%) of our cohort while 147 (56.5%) had anxiety. Similar rates of adherence were seen amongst males (49.1%) and females (50.9%). The highest level of adherence was seen in the 61 to 75 years age group (34.9%) and in those with university-level education (30.6%). We also found a statistically significant association between adherence to antihypertensive medications with anxiety (p-value=0.048). Moreover, in the hypertensive crisis group, 40.7% of the patients were adherent to their antihypertensive medications while 54.8% were found to be non-adherent, with the p-value reaching statistical significance (p-value=0.028).

Conclusion: We found a higher rate of adherence (64%) in this inpatient hypertensive population as compared to previous studies in our population. We also found that non-adherence is a risk factor for the development of a hypertensive crisis. Therefore, at clinic visits, physicians should assess their patient’s adherence to antihypertensive medications to prevent the development of a hypertensive crisis.

## Introduction

Hypertension has been established as a major risk factor for cardiovascular disease (CVD) which is the leading cause of death worldwide, accounting for an estimated 17.3 million deaths per year [1]. The global prevalence of hypertension is also on the rise. In 2010, 31.1% of people worldwide had hypertension, with 75% of those people residing in low and middle-income countries [2]. In Pakistan, a metanalysis estimated the prevalence of hypertension to be 26.3%, which is only expected to rise with the increase in urbanization and epidemiological transitions [3]. It is estimated that 1% of hypertensive patients will develop a hypertensive crisis owing to poorly controlled hypertension which could be due to non-compliance or non-adherence to antihypertensive medication [4].

Medication adherence is defined as 'the extent to which the medication-taking behavior of a patient corresponds with agreed recommendations from a health care provider' [5]. According to WHO, non-adherence to antihypertensive medications is a major cause of poorly controlled hypertension and almost 50% to 70% of patients adhere poorly to their prescribed treatment [5]. In terms of adherence in our population, a study showed that 38.3% of the Pakistani population was non-adherent to antihypertensive medications [6]. While non-adherence to medications results in undesirable health outcomes with higher rates of morbidity and mortality, it also leads to greater utilization and wastage of health resources [7,8].

Non-adherence also increases the risk of hypertensive crisis. The strongest predictor for the development of a hypertensive crisis, according to a prospective longitudinal study, was non-adherence to antihypertensive treatment [9]. Other studies have reproduced similar results, strengthening the association between the two entities [9-11]. At the patient level, several factors have been identified that lead to non-adherence. These include lack of symptoms, treatment-related adverse effects, polypharmacy, forgetfulness, comorbidities including depression, and sociodemographic factors such as age, sex, education level, employment, and family support [12].

There are numerous studies in the literature that examine the causes of non-adherence in Pakistani hypertensive patients, but there is a dearth of information on the relationship between non-adherence and hypertensive crisis. The aim of our study was thus to estimate the frequency of adherence to antihypertensive medications overall and to analyze if there is an association between adherence to antihypertensive therapy and hypertensive crisis.

## Materials and methods

We conducted this cross-sectional study on 290 patients admitted at Aga Khan University Hospital (AKUH), Karachi, Pakistan, between July 2020 and March 2022. The ethics committee of the AKUH approved the study (approval no. 2020-3414-10233). The AKUH serves a wide range of patients from across Pakistan as it is one of the country's major Joint Commission International-accredited tertiary care university hospitals (740 beds). We included all adult patients aged more than or equal to 18 years of age with uncontrolled hypertension with systolic blood pressure >140 and diastolic blood pressure >90 who were admitted to the medicine ward. We excluded all patients admitted with raised blood pressure resulting from secondary causes such as stressors, kidney diseases, stimulant drug-induced, and endocrine disorders. Systolic blood pressure over 180 mmHg or diastolic blood pressure over 120 mmHg, with or without end-organ damage, was labeled as a hypertensive crisis [4]. At least two persistently elevated blood pressure readings confirmed a hypertensive crisis.

The adherence to antihypertensive medications was assessed using the Morisky Green Levine (MGL) medication adherence scale. Developed in 1986 from an original five-item tool, the MGL scale is a four-item standard tool used to assess medication adherence [13]. The MGL scale comprises four questions, each of which is asked in the reverse direction to minimize social desirability bias [13]. Each item on the MGL scale has a yes or no response with 0 points given to 'yes' and 1 point to 'no' [13]. By asking questions on forgetfulness or carelessness, the MGL scale evaluates the unintentional aspects of medication non-adherence, and the two questions on cessation of prescribed medications when feeling better or worse address the intentional aspects of medication non-adherence [13]. In our study, we dichotomized the full score on the MGL scale into two groups; those who scored 2-4 on the MGL scale were classified as adherent and those with scores of 0-1 were grouped as non-adherent [14].

Data on patients’ baseline demographic characteristics and comorbid conditions were also collected. Baseline characteristics included age, gender, education, employment status, marital status, obesity, alcohol abuse, and smoking. Comorbid conditions included diabetes, coronary artery disease, depression and anxiety, ischemic heart disease, and a history of cerebrovascular disease. The duration of hypertension was also recorded. Patients were deemed to have diabetes mellitus if they were previously known diabetics, had hemoglobin A1c (HbA1c) of 6.5% or above, or had a random plasma glucose value of 200 mg/dl (11.1 mmol/l), or a fasting plasma glucose value of 126 mg/dl (7.0 mmol/l) [15]. Patients who had any history of stable ischemic heart disease or acute coronary syndrome (ST-elevation myocardial infarction (STEMI), non-ST elevation myocardial infarction (NSTEMI), or unstable angina) were diagnosed as suffering from coronary artery disease [16]. The hospital anxiety and depression scale (HADS) was used to assess anxiety and depression in the patients [17]. Alcohol abuse was present if the daily alcohol consumption exceeded three drinks a day (corresponding to ~30 g/day) [18]. Patients who had stopped smoking at least one year before the study was characterized as past smokers while those currently smoking were further categorized according to the number of cigarettes smoked per day (light=1 to 9, moderate=10 to 19, heavy=more than 20) [19]. Patients admitted with symptoms of ischemic or hemorrhagic infarcts or those with a previous history of cerebrovascular accidents were recorded as having cerebrovascular disease [20]. Using the BMI values defined for the Indo-Asian population, patients with a BMI of greater than 23.0 were considered obese [21]. A sample size of 290 participants was targeted to estimate the prevalence of non-adherence of 48.6% and a significance level of 0.05. The sample size estimate was based on using a power of at least 80%.

Statistical Package for Social Sciences (SPSS) version 19.1® (IBM Corp., Armonk, NY, USA) was used for analysis. Mean and standard deviation was calculated for quantitative variables and frequency and percentage for qualitative variables. Student’s t-test was used to compare quantitative variables and the chi-square test was used to compare qualitative variables. The Fischer exact test was used when the cell count was less than 5. 

## Results

In our study, a total of 260 participants fulfilled the inclusion criteria and were enrolled. Around 167 (64%) of our cohort were adherent to their medications while 93 (36%) were non-adherent. Of the study population, 48.5% were males, and 51.5% were females. The majority of patients were in the 61 to 75 years age group (37.2%), were non-smokers (80.2%), and had secondary or university education (63.4%). The most common co-morbid condition after hypertension was found to be diabetes mellitus (54.8%). Out of the total, 146 (64.6%) of the patients were on a single anti-hypertensive agent. Depression, as calculated by the HADS scale, was seen in 133 (51.2%) of our cohort and 147 (56.5%) had anxiety. The baseline characteristics of hypertensive patients are shown in Table 1. When stratified by adherence, similar rates were seen amongst the male (49.1%) and the female group (50.9%). The highest level of adherence was seen in the 61 to 75 years age group (34.9%). Our results also show that there were statistically significant associations between adherence to antihypertensive medications with anxiety (p-value=0.048).

**Table 1:** Baseline characteristics of hypertensive patients admitted to a tertiary care hospital in Karachi, Pakistan HADS: Hospital anxiety and depression scale, DM: Diabetes mellitus, CKD: Chronic kidney disease, IHD: Ischemic heart disease

	Total	Adherence	Non-adherence	P-value
	N = 260 (100%)	n = 67 (64%)	n = 93 (36%)	
Gender				0.782
Male	126 (48.5)	82 (49.1)	44 (47.3)	
Female	134 (51.5)	85 (50.9)	49 (52.7)	
Age				0.078
21-45 years	37 (14.3)	24 (14.5)	13 (14.1)	
46-60 years	89 (34.5)	54 (32.5)	35 (38.0)	
61-75 years	96 (37.2)	58 (34.9)	38 (41.3)	
76-95 years	36 (14.0)	30 (18.1)	6 (6.5)	
Employment				0.76
Yes	67 (25.8)	42 (25.1)	25 (26.9)	
No	193 (74.2)	125 (74.9)	68 (73.1)	
Education Level				0.007
None	36 (13.8)	23 (13.8)	13 (14)	
Primary	54 (20.8)	43 (25.7)	11 (11.8)	
Secondary	82 (31.5)	44 (26.3)	38 (40.9)	
College/university	83 (31.9)	56 (33.5)	27 (29.0)	
HADS Depression				0.089
Yes	133 (51.2)	92 (55.1)	41 (44.1)	
No	127 (48.8)	75 (44.9)	52 (55.9)	
HADS Anxiety				0.048
Yes	147 (56.5)	102 (61.1)	45 (48.4)	
No	113 (43.5)	65 (38.9)	48 (51.6)	
Marital Status				0.283
Married	208 (80)	129 (77.2)	79 (84.9)	
Never married	9 (3.5)	5 (3.0)	4 (4.3)	
Widowed	40 (15.4)	31 (18.6)	9 (9.7)	
Comorbidities				
DM	142 (54.8)	87 (52.4)	55 (59.1)	0.296
CKD	52 (20.3)	33 (20.2)	19 (20.4)	0.972
IHD	74 (28.7)	42 (25.5)	32 (34.4)	0.127
Cerebrovascular accident	30 (11.5)	19 (11.4)	11 (11.8)	0.913
Smoking Status				0.992
Non-smoker	207 (80.2)	132 (80)	75 (80.6)	
Past smoker	31 (12.0)	20 (12.1)	11 (11.8)	
Current	20 (7.8)	13 (7.9)	7 (7.5)	
Number of Hypertensive Medications				0.219
1	146 (64.6)	95 (65.5)	51 (63)	
2	64 (28.3)	44 (30.3)	20 (24.7)	
3	11 (4.9)	4 (2.8)	7 (8.6)	
More than or equal to 4	2 (0.9)	1 (0.7)	1 (1.2)	
Obesity				0.891
Obese	171 (78.4)	111 (78.7)	60 (77.9)	
Non-obese	47 (21.6)	30 (21.3)	17 (22.1)	
Duration of Hypertension				0.674
Less than 10 years	117 (47.2)	78 (48.1)	39 (45.3)	
More than 10 years	131 (52.8)	84 (51.9)	47 (54.7)	
Hypertensive crisis	119 (45.8)	68 (40.7)	51 (54.8)	0.028

Table 2 highlights the comparison of individual items in adherence scale between patients who are adherent and non-adherent to antihypertensives.

**Table 2:** Comparison of individual items in adherence scale between patients who are adherent and non-adherent to anti-hypertensives at a tertiary hospital in Karachi, Pakistan *Patients who scored 2-4 on the Moriskly Green Levine scale are considered adherent while patients who scored 0-1 are considered non-adherent

Questionaire & response	Overall N (%)	Patients who are adherent to antihypertensive medications* n=167 (64%)	Patients who are non-adherent to hypertensive medications* n=93 (36%)	p-value
Do you ever forget to take your medicines? Yes	107 (41.2)	28 (16)	79 (84)	<0.001
Are you careless at times about taking your medicines? Yes	113 (4.5)	27 (16.2)	86 (92.5)	<0.001
Do you sometimes stop taking your medicines when you feel better? Yes	129 (49.6)	44 (26.3)	85 (91.4)	<0.001
Sometimes if you feel worse when you take the medicine, do you stop taking it? Yes	139 (53.5)	53 (31.7)	86 (92.5)	<0.001

Table 3 shows the comparison of individual items in the adherence scale in patients with and without hypertensive crisis at a tertiary hospital in Karachi, Pakistan. There was no difference in individual components of adherence in patients with or without hypertensive crisis.

**Table 3:** Comparison of individual items in adherence scale in patients with and without hypertensive crisis at a tertiary hospital in Karachi, Pakistan * Hypertensive crisis is described as systolic blood pressure more than or equal to 180 mmHg or diastolic blood pressure more than or equal to 120 mmHg

Questionaire & response	Overall N (%)	Patients with hypertensive crisis*	Patients without hypertensive crisis	p-value
Do you ever forget to take your medicines? Yes	107 (41.2)	46 (38.7)	61 (43.3)	0.452
Are you careless at times about taking your medicines? Yes	113 (43.5)	59 (49.6)	54 (38.3)	0.067
Do you sometimes stop taking your medicines when you feel better? Yes	129 (49.6)	66 (55.5)	63 (44.7)	0.087
Sometimes if you feel worse when you take the medicine, do you stop taking it? Yes	139 (53.5)	62 (52.1)	77 (54.6)	0.686

**Figure 1:**
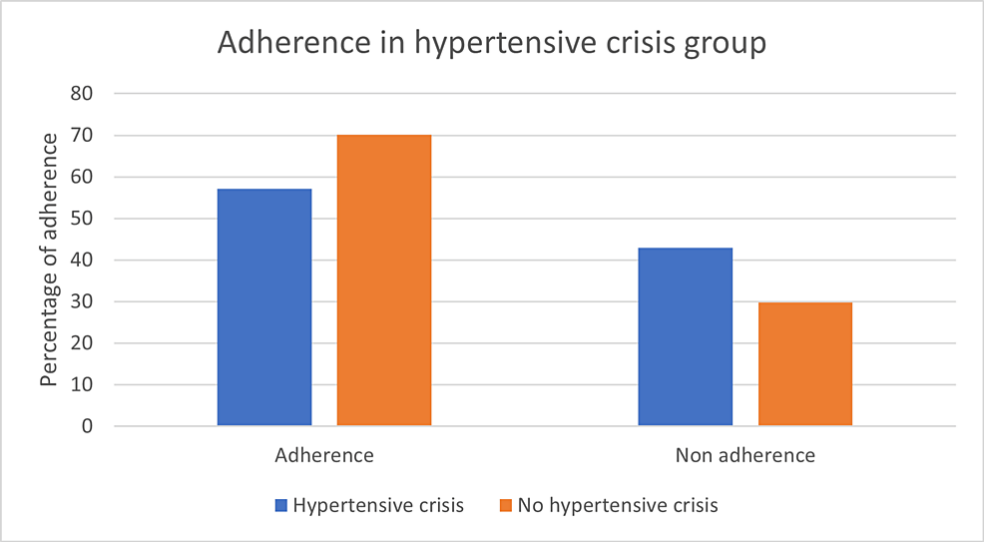
Adherence status and hypertensive crisis p-value=0.028

In the hypertensive crisis group, 68 (40.7%) patients were adherent to their anti-hypertensives while 51 (54.8%) were found to be non-adherent, with the p-value reaching statistical significance (p=0.028). This association between hypertensive crisis and adherence is illustrated in Figure 1. 

## Discussion

We found that 64% of our patients were adherent to their prescribed antihypertensive therapy while 36% were non-adherent. Furthermore, we also found an association between adherence to antihypertensives and hypertensive crisis. Overall, in Asia, a metaanalysis estimated the prevalence of non-adherence to antihypertensive medication to be about 48% [22]. On further stratification, the prevalence of non-adherence was found to be 48% in South Asia, 45% in East Asia, and 41% in the Middle East [22]. The same metanalysis concluded that in low and lower-middle-income countries, the rate of non-adherence was 50% which was higher when compared to upper-middle and high-income countries where the rate of non-adherence was 37% [22]. In previous studies from Pakistan, the rates of non-adherence range from 51% to 67% [23,24]. Compared to other regions in Pakistan and our own report on compliance to antihypertensives in which 46% of patients were non-compliant [25], the nonadherence to antihypertensives in our study is lower i.e., 36%.

Our results are, however, comparable to the non-adherence reported by a metanalysis in Western countries (38%) with a higher rate (43%) in non-Western countries [26]. The reason for this relatively lower non-adherence rate as compared to other non-Western countries could be due to the fact that this study was mainly on inpatients from a tertiary care center indicating that they might be having severe illnesses requiring admissions. Secondly, we receive a significant number of patients from middle and high socioeconomic status and are likely to have better knowledge about hypertension and medication adherence. Several other factors have been identified that result in medication non-adherence. At the patient level, factors such as forgetfulness, communication barriers, patient knowledge, and preferences are significant [22,23]. Moreover, therapy-related adverse effects and frequency of dosing regimens also play a role in non-adherence [22]. Unaffordability, unemployment, and financial constraints are other factors cited by various studies [22].

According to our findings, patients who did not take their antihypertensive medications were more likely to develop hypertensive crises. This observation is also supported by previous studies. Wallbach et al. used biochemical urine analysis to assess this relationship and discovered that 58% of patients with hypertensive crisis had poor adherence (p=0.01) [27]. Similarly, Martin et al. included 452 hypertensive crisis patients in their study and reported similar findings [28]. Non-adherence to antihypertensive medications was also found to be the single most important factor in the development of hypertensive crisis by Saguner et al. (hazard ratio 5.88, p=0.01) [9]. However, a systematic review of 11,783 patients found that non-adherence to hypertensive medications was not associated with the development of hypertensive crisis (odds ratio (OR) 0.939, 95% confidence interval (CI) 0.647,1.363) [29]. These differences can be explained by the different methods used to quantify adherence. Some studies used a validated questionnaire to assess adherence, while others measured antihypertensive medication levels in blood or urine. The reason for non-adherence in patients with a hypertensive crisis could be that they are more likely to have anxiety as reported in our previous study that 59% of patients with hypertensive crisis had anxiety, although the association could not reach statistical significance [30].

There are some limitations to this study that need to be highlighted. We used self-reported questionnaires to measure treatment adherence which risks the potential for recall as well as social desirability bias. Moreover, our study was carried out at a single center with a small sample size which limits the generalizability of our findings.

## Conclusions

In conclusion, we found a higher rate of adherence in this inpatient hypertensive population as compared to previous studies. We also found that non-adherence is a risk factor for the development of hypertensive crises. At clinic visits, therefore, physicians should use the opportunity to assess their patient’s adherence to antihypertensive medications to prevent the development of a hypertensive crisis which is a potentially life-threatening complication of uncontrolled blood pressure.
